# The Use of Instrumental Variables in Peer Effects Models†

**DOI:** 10.1111/obes.12299

**Published:** 2019-02-04

**Authors:** Stephanie von Hinke, George Leckie, Cheti Nicoletti

**Affiliations:** ^1^ Department of Economics University of Bristol 8 Woodland Road Bristol BS8 1TN UK; ^2^ Erasmus School of Economics Erasmus University Rotterdam Rotterdam The Netherlands; ^3^ Institute for Fiscal Studies London UK; ^4^ Centre for Multilevel Modelling University of Bristol Bristol UK; ^5^ Department of Economics and Related Studies University of York Heslington York YO10 5DD UK; ^6^ ISER University of Essex Colchester UK

## Abstract

Instrumental variables are often used to identify peer effects. This paper shows that instrumenting the ‘peer average outcome’ with ‘peer average characteristics’ requires the researcher to include the instrument at the individual level as an explanatory variable. We highlight the bias that occurs when failing to do this.

## 1 Introduction

Many papers in economics provide empirical evidence on the causal effect of peers on individual outcomes using an instrumental variable (IV) approach. They usually consider linear in mean regressions of an individual outcome on the corresponding average outcome of peers and a set of individual explanatory variables. They may then instrument the average outcome of peers with the peer average of certain characteristics.^1^

As in any other standard linear regression, the IV estimator consistently estimates the causal peer effect if the instruments are as good as randomly assigned (independence), irrelevant in explaining the individual outcome except through the average peers’ outcome (exclusion), and relevant in explaining the endogenous outcome averaged across peers (relevance).^2^

The contribution of this paper is to highlight a subtle, but important implication of the relevance assumption, something not explicitly recognized in this literature: the individual variable, say *x*, whose peer average, say $\overset{¯}{x}$, is used to instrument the peer average outcome $\overset{¯}{y}$ must be included as an individual explanatory variable of the dependent variable *y*. The idea is simple: if $\overset{¯}{x}$ is a valid instrument for $\overset{¯}{y}$, then *x* must also be related to *y* at the individual level. We show that failing to include the individual variable leads to inconsistent estimates. The only case when consistency holds is if peers are randomly allocated across individuals. However, even if peers are randomly allocated *within* clusters (e.g. schools) but not *across* clusters, the inclusion of cluster fixed effects – a necessity as randomization takes place within clusters – renders the estimates inconsistent.^3^

While most applications of peer effects that use IV do include the instrument at the individual level and therefore avoid the inconsistency and bias described here, a number of papers have not done so. More generally, we have found no discussion of this issue in the literature. Given the widespread use of IV in peer effects models, we argue that it is important to raise awareness of this among both econometricians and applied researchers.

## 2 The peer effects model

As the consistency of the instrumental variable estimation of a peer effect depends on whether cluster fixed effects are controlled for, we discuss both cases separately, and end with a formal proof of the asymptotic bias. To better clarify what we mean by peers and clusters, consider the case where the peer group is defined by the classmates within schools, then the peer effect is the effect of the classmates, while the cluster fixed effect is the school fixed effect.

### The case without cluster fixed effects

We follow the existing literature that almost exclusively specifies a linear‐in‐mean peer effects model and consider the following specification

(1) y=Wyρ+u,

where **y** is the *N*×1 vector of the individual outcome, **W** is an *N*×*N* row‐standardized weight matrix describing the social ties between individuals, *ρ* is the scalar peer effect parameter and **u** is the residual error vector.^4^ Model (1) does not include the intercept but there is no loss of generality as long as all variables are expressed as deviations from their means. Furthermore, as we discuss below, the model can easily be adjusted to account for additional explanatory variables.

The instruments for **Wy** are defined as the peer average of characteristics **X**, i.e. **WX**. These must satisfy *independence*,* exclusion* and *relevance*. *Exclusion* assumes that the instruments **WX** only affect **y** through **Wy**, i.e. that there is zero correlation between the error term in model (1) and **WX**, or *corr*(**WX**,**u**)=0; *relevance* requires the instruments to explain variation in **Wy**, i.e. that *corr*(**Wy**,**WX**)≠0. The IV estimation of the peer effect *ρ*, which we refer to as ${\hat{\rho }}_{IV0}$ is then given by:

(2) $${\hat{\rho }}_{IV0}={\left[{\left(\mathrm{Wy}\right)}^{\prime }P_{\mathrm{WX}}\left(\mathrm{Wy}\right)\right]}^{-1}{\left(\mathrm{Wy}\right)}^{\prime }P_{\mathrm{WX}}y,$$

where **P**_**WX**_ is the projection matrix [(**WX**)[(**WX**)^′^(**WX**)]^**−1**^(**WX**)^′^]. The IV estimator ${\hat{\rho }}_{IV0}$ is equivalent to a 2‐stage least squares (2SLS) estimator where the first stage is the ordinary least squares (OLS) regression of **Wy** on **WX**, and the second stage is the OLS regression of **y** on the prediction of **Wy** obtained from the first stage, i.e. [**P**_**WX**_(**Wy**)] (see e.g. Cameron and Trivedi, 2005).

The peer effects literature that adopts this IV approach assumes that the individual outcome **y** is not directly affected by peers’ average characteristics **WX**, but they generally do not make any explicit assumption on whether the individuals’ characteristics **X** directly affect **y**. Appendix A shows that under the relevance and exclusion assumptions, it follows that **X** directly affects **y**, and hence model (1) is misspecified because it omits **X** from the explanatory variables.^5^ In other words, **X** should be included as explanatory variables in model (1):

(3) y=Wyρ+Xγ+ϵ,

where we still omit the constant and assume that all variables, including **X**, are expressed in deviation from their mean. We therefore refer to equation (3) as the true model.^6^ By replacing **y** in equation (2) with the right‐hand side of equation (3), we can show that the estimator ${\hat{\rho }}_{IV0}$ in equation (2) is inconsistent:

(4) $${\hat{\rho }}_{IV0}=\rho +{\left[{\left(\mathrm{Wy}\right)}^{\prime }P_{\mathrm{WX}}\left(\mathrm{Wy}\right)\right]}^{-1}{\left(\mathrm{Wy}\right)}^{\prime }P_{\mathrm{WX}}\left(X\gamma +\epsilon \right).$$

Denoting [**P**_**WX**_(**Wy**)] with $\left(\mathrm{WX}\right)\hat{\lambda }$, where $\hat{\lambda }$ is the OLS estimator of the coefficients of **WX** in the first stage regression of **Wy** on **WX**, and taking the probability limit, we obtain

(5) $$p-\text{lim}\;{\hat{\rho }}_{IV0}=\rho +{\left(\lambda^{\prime }E\left({\left(\mathrm{WX}\right)}^{\prime }\left(\mathrm{WX}\right)\right)\lambda \right)}^{-1}\lambda^{\prime }E\left({\left(\mathrm{WX}\right)}^{\prime }\left(X\gamma +\epsilon \right)\right),$$

where $\lambda =p-\text{lim}\;\hat{\lambda }$, which is the vector of the true slope coefficients of **WX** in the linear regression of **Wy** on **WX**. This shows that the IV estimation is consistent if and only if *E*((**WX**)^′^(**X*γ***+***ε***))=0. We discuss this separately as *E*((**WX**)^′^ **X**)=0 and *E*((**WX**)^′^ ***ε***)=0. The latter is the main assumption imposed by empirical studies that estimate peer effects by instrumenting the peer average **Wy** with **WX**. The condition *E*((**WX**)^′^ **X**)=0 is satisfied when peers are randomly allocated across individuals. If, instead, peers are randomly allocated *within* clusters, but not *across* clusters, **X** may have a different distribution across these clusters, leading to *E*((**WX**)^′^ **X**)≠0 and potentially biasing the estimation. For example, university classmates can be randomly chosen from the students enrolled in a specific degree but not from other degrees, or university roommates can be randomly chosen within a college but not across colleges (see e.g. the review by *Sacerdote* 2001). Because students do not randomly select into different colleges or degrees, peers (i.e. class or roommates) are not necessarily randomly allocated across such clusters.

Nevertheless, this potential inconsistency can be solved by controlling for the individual variables **X** as in model (3), and adopting the following IV estimation

(6) $${\hat{\rho }}_{IV1}={\left[{\left(M_{X}\left(\mathrm{Wy}\right)\right)}^{\prime }P_{M_{X}\left(\mathrm{WX}\right)}\left(M_{X}\left(\mathrm{Wy}\right)\right)\right]}^{-1}{\left(M_{X}\left(\mathrm{Wy}\right)\right)}^{\prime }P_{M_{X}\left(\mathrm{WX}\right)}\left(M_{X}y\right),$$

where **M**_**X**_=**I**−**X**(**X** ^′^ **X**)^−1^ **X**,** I** is the identity matrix, and $P_{M_{X}\left(\mathrm{WX}\right)}$ is the projection matrix $\left(M_{X}\left(\mathrm{WX}\right)\right){\left[{\left(M_{X}\left(\mathrm{WX}\right)\right)}^{\prime }\left(M_{X}\left(\mathrm{WX}\right)\right)\right]}^{-1}{\left(M_{X}\left(\mathrm{WX}\right)\right)}^{\prime }$. The estimator ${\hat{\rho }}_{IV1}$ is a standard two‐stage least squares estimation applied to model (3) transformed by premultiplying all variables by **M**_**X**_:

(7) $$M_{Xy}=M_{X\mathrm{Wy}}\rho +M_{X}\epsilon ,$$

with instruments **M**_**X**(**WX**)_, i.e. the original instruments (**WX**) premultiplied by **M**_**X**_. Note that transforming model (3) by premultiplying each variable by **M**_**X**_ is equivalent to replacing each variable with the residual from the regression of the variable itself on the explanatory variables **X**. By applying the Frisch–Waugh theorem, we can prove that the above transformation does not affect the estimation of the peer effect *ρ*.

We refer to the estimation of ${\hat{\rho }}_{IV1}$ as *IV approach 1*, i.e. the approach that *includes* the instrument at the individual level; we refer to the estimation of ${\hat{\rho }}_{IV0}$ as *IV approach 0*, i.e. the estimation approach that *omits* the instrument at the individual level.

### The case with cluster fixed effects

In applied work, peers are sometimes randomized *within* but not *across* clusters. For example, class peers are often randomly chosen from the set of children enrolled in a school, but because children do not randomly sort into schools, the distribution of individual characteristics **X** is likely to differ between schools, leading to *E*((**WX**)^′^ **X**)≠0 and potentially biasing the instrumental variable estimation ${\hat{\rho }}_{IV0}$. Because randomization in such cases is within schools, analyses of these experiments necessarily include school (or cluster) fixed effects. We now show that failing to include the instrument at the individual level leads to inconsistent estimation of the peer effect in models with cluster fixed effects, even in cases where peers are randomized.

Consider the following fixed effects model:

(8) y=Wyρ+Dδ+ν,

where, **D** is the *N*×*J* matrix of binary cluster indicators, *J* is the number of clusters, ***δ*** is the corresponding vector of fixed effects and ***ν***=**X*γ***+**e**. Applying cluster–mean deviations, we can rewrite equation (8) as follows:

(9) $$y_{*}={\left(\mathrm{Wy}\right)}_{*}\rho +\nu_{*},$$

where the subscript * indicates that the variable is premultiplied by the orthogonal projection matrix **M**_**D**_=**I**−**D**(**D** ^′^ **D**)^−1^ **D** ^′^: y_*_=**M**_**D**_ **y**, (**Wy**)_*_=**M**_**D**_(**Wy**), and $\nu_{*}=X_{*}\gamma +e_{*}=M_{D}\nu$. In other words, model (9) is equal to model (8) with the variables transformed to indicate deviations from their cluster means (i.e. a within‐cluster transformation).

Using the instrument (**WX**)_*_, the IV estimator that fails to control for the individual variables $X_{*}$, i.e. *IV approach 0*, can be written as

(10) $${\hat{\rho }}_{*IV0}={\left[{\left(\mathrm{Wy}\right)}_{*}^{\prime }P_{{\mathrm{WX}}_{*}}{\left(\mathrm{Wy}\right)}_{*}\right]}^{-1}{\left(\mathrm{Wy}\right)}_{*}^{\prime }P_{{\mathrm{WX}}_{*}}y_{*},$$

where $P_{{\mathrm{WX}}_{*}}$ is the projection matrix $\left[{\left(\mathrm{WX}\right)}_{*}{\left({\left(\mathrm{WX}\right)}_{*}^{\prime }{\left(\mathrm{WX}\right)}_{*}\right)}^{-1}{\left(\mathrm{WX}\right)}_{*}^{\prime }\right]$.^7^ With $\nu_{*}=X_{*}\gamma +e_{*}$, this converges in probability to

(11) $$p-\text{lim}\;{\hat{\rho }}_{*IV0}=\rho +{\left[\lambda_{*}^{\prime }E\left({\left(\mathrm{WX}\right)}_{*}^{\prime }{\left(\mathrm{WX}\right)}_{*}\right)\lambda_{*}\right]}^{-1}\lambda_{*}^{\prime }E\left({\left(\mathrm{WX}\right)}_{*}^{\prime }\left(X_{*}\gamma +e_{*}\right)\right),$$

where $\lambda_{*}=p-\text{lim}{\left({\left(\mathrm{WX}\right)}_{*}^{\prime }{\left(\mathrm{WX}\right)}_{*}\right)}^{-1}{\left(\mathrm{WX}\right)}_{*}^{\prime }{\left(\mathrm{Wy}\right)}_{*}$ is the effect of the instruments (**WX**)_*_ on the peer average outcome (**Wy**)_*_. Hence, consistency of equation (11) requires that $E({\left(\mathrm{WX}\right)}_{*}^{\prime }\left(X_{*}\gamma +e_{*}\right))=0$. Under random assignment of peers across individuals, the individual vector of characteristics **X** is uncorrelated with **WX**. This is because **WX** is the peer average excluding the individual herself, and the random assignment of peers implies that **X** is identically and independently distributed (i.i.d.) across individuals. Nevertheless, random assignment within clusters does not imply a zero correlation between the *transformed* variables $X_{*}$ and (**WX**)_*_, i.e. between the within‐cluster deviations of **X** and **WX**, and therefore $E({\left(\mathrm{WX}\right)}_{*}^{\prime }X_{*})=0$ does not necessarily hold.

To prove this and without loss of generality, we consider a scalar exogenous variable *x*_*i*_ and the corresponding scalar instrumental variable ${\overset{¯}{x}}_{-i}^{p}$, which is the usual peer average of *x* excluding individual *i*. Then the within‐cluster deviations of *x*_*i*_ and ${\overset{¯}{x}}_{-i}^{p}$ are equal to $\left(x_{i}-{\overset{¯}{x}}_{i}^{c}\right)$ and $\left({\overset{¯}{x}}_{-i}^{p}-{\overset{¯}{\overset{¯}{x}}}_{i}^{\mathrm{pc}}\right)$, respectively, where ${\overset{¯}{x}}_{i}^{c}$ is the cluster average of *x*_*i*_ including the individual *i*, ${\overset{¯}{\overset{¯}{x}}}_{i}^{\mathrm{pc}}=\sum_{j=1}^{n_{c,\;i}}{\overset{¯}{x}}_{-j}^{p}/n_{c,\;i}$ is the cluster average of the peer average of all members in the cluster of individual *i*, and $n_{c,\;i}$ is the number of members in this cluster including individual *i*. By excluding the very unlikely case where individuals interact exclusively with peers who do not belong to their cluster, we can prove that $\left(x_{i}-{\overset{¯}{x}}_{i}^{c}\right)$ and $\left({\overset{¯}{x}}_{-i}^{p}-{\overset{¯}{\overset{¯}{x}}}_{i}^{\mathrm{pc}}\right)$ are correlated. Let us consider an individual *k* who is a (randomly assigned) peer of individual *i* belonging to the same cluster; then her observed characteristic *x*_*k*_ will contribute to both the cluster and the peer averages of individual *i*, ${\overset{¯}{x}}_{i}^{c}$ and ${\overset{¯}{x}}_{-i}^{p}$ respectively. Hence both $\left(x_{i}-{\overset{¯}{x}}_{i}^{c}\right)$ and $\left({\overset{¯}{x}}_{-i}^{p}-{\overset{¯}{\overset{¯}{x}}}_{i}^{\mathrm{pc}}\right)$ will be correlated with *x*_*k*_ and therefore $corr(\left(x_{i}-{\overset{¯}{x}}_{i}^{c}\right)\left({\overset{¯}{x}}_{-i}^{p}-{\overset{¯}{\overset{¯}{x}}}_{i}^{\mathrm{pc}}\right))\neq 0$, *despite* random assignment of peers.

Generalizing of the above proof to multivariate instruments, we can see that random assignment within clusters does not imply a zero correlation between $X_{*}$ and (**WX**)_*_. Ultimately, this implies that the instrumental variables (**WX**)_*_ will be correlated with $\nu_{*}=X_{*}\gamma +e_{*}$, i.e. the error term in equation (9), biasing the instrumental variable estimation. Note that the bias is induced by the within transformation: it exists even if the *untransformed* instrumental variable **WX** is unrelated to the *untransformed* errors ***ν***.^8^ Avoiding this bias is possible by including the instruments at the individual level, $X_{*}$, in the peer effects model, as in *IV Approach 1*,^9^ considering the following model

(12) $$y_{*}={\left(\mathrm{Wy}\right)}_{*}\rho +X_{*}\gamma +e_{*}.$$

The IV estimator for the peer effect can then be written as

(13) $${\hat{\rho }}_{*IV1}={\left[{\left(M_{X_{*}}{\left(\mathrm{Wy}\right)}_{*}\right)}^{\prime }P_{M_{X_{*}}{\left(\mathrm{WX}\right)}_{*}}\left(M_{X_{*}}{\left(\mathrm{Wy}\right)}_{*}\right)\right]}^{-1}{\left(M_{X_{*}}{\left(\mathrm{Wy}\right)}_{*}\right)}^{\prime }P_{M_{X_{*}}{\left(\mathrm{WX}\right)}_{*}}M_{X_{*}}y_{*},$$

where $M_{X_{*}}=I-X_{*}{\left(X_{*}^{\prime }X_{*}\right)}^{-1}X_{*}^{\prime }$, and $P_{M_{X_{*}}{\left(\mathrm{WX}\right)}_{*}}$ is the projection matrix on the space generated by the columns of $M_{X_{*}}{\left(\mathrm{WX}\right)}_{*}$.^10^ By replacing y_*_ in equation (13) with the right‐hand side of equation (12), we can show that ${\hat{\rho }}_{*IV1}$ converges in probability to *ρ* if $E({\left(\mathrm{WX}\right)}_{*}^{\prime }e_{*})=0$.

### Asymptotic bias

We next characterize the asymptotic bias. For this, we assume that equation (12) represents the true model (or equation (3) for the case without cluster fixed effects). However, if the true model specifies **y** as some other function of the instrument at the individual level (e.g. **X** ^**2**^ or **ln**(**X**)), the asymptotic bias will be different and hence, our derivations only refer to the case where **X** enters the specification in an additively separable way.

Assuming $E({\left(\mathrm{WX}\right)}_{*}^{\prime }e_{*})=0$, the asymptotic bias of the estimator ${\hat{\rho }}_{*IV0}$ is given by

$${\left[\lambda_{*}^{\prime }E\left({\left(\mathrm{WX}\right)}_{*}^{\prime }{\left(\mathrm{WX}\right)}_{*}\right)\lambda_{*}\right]}^{-1}\lambda_{*}^{\prime }E\left({\left(\mathrm{WX}\right)}_{*}^{\prime }X_{*}\right)\gamma ;$$

as shown by equation (11) above. Nevertheless, it is difficult to predict its sign and magnitude because it depends on (i) the effect of the instrument at the individual level on the individual outcome, i.e. ***γ***, (ii) the effect of the instruments on the peer average outcome λ_*_, (iii) $E({\left(\mathrm{WX}\right)}_{*}^{\prime }X_{*})$, and (iv) on $E({\left(\mathrm{WX}\right)}_{*}^{\prime }{\left(\mathrm{WX}\right)}_{*})$. Nevertheless, we can characterize the asymptotic bias in the case with one instrument as shown in the following Proposition.

Proposition 1 Let us assume that the following conditions hold.

A1 **Correct model specification:** The true model for *y*_*i*_ is given by
  (14) $$y_{i}={\overset{¯}{y}}_{-i}^{p}\rho +x_{i}\gamma +d_{i}\delta +e_{i},$$
  where the subscript *i*=1,…,*N* denotes individuals; *y*_*i*_ and *x*_*i*_ are demeaned; ${\overset{¯}{y}}_{-i}^{p}$ is the peer average of *y* excluding individual *i*;* x*_*i*_ is a scalar exogenous variable; **d**_*i*_ is the 1×*J* vector of cluster indicators; *J* is the number of clusters; *e*_*i*_ is an idiosyncratic error uncorrelated with the explanatory variables except for the endogenous variable ${\overset{¯}{y}}_{-i}^{p}$; and (*y*_*i*_,*x*_*i*_,*e*_*i*_) are i.i.d. with means zero and variances $\sigma_{x}^{2}$, $\sigma_{y}^{2}$ and $\sigma_{e}^{2}$.
A2 **Three‐level hierarchical balanced data structure:** Individuals (level 1) are nested within peer groups (level 2), which are nested within clusters (level 3). The data are balanced in the sense that all peer groups and all clusters have the same number of individuals, which we denote with $n_{p}$ and $n_{c}$ respectively.
A3 **Random assignment:** Peers are randomly assigned across individuals within clusters.
A4 **Exogeneity of the instrument:** There is no correlation between the deviation from the cluster mean of the error term, $e_{i,\;*}=e_{i}-{\overset{¯}{e}}_{i}^{c}$, and of the instrument, ${\overset{¯}{x}}_{-i,\;*}^{p}={\overset{¯}{x}}_{-i}^{p}-\sum_{j=1}^{n_{c}}{\overset{¯}{x}}_{-j}^{p}/n_{c}$, where the sum is over all individuals belonging to the same cluster as individual *i*.

Then the asymptotic bias in the IV estimation that uses ${\overset{¯}{x}}_{-i}^{p}$ to instrument for ${\overset{¯}{y}}_{-i}^{p}$ but omits to include *x*_*i*_ among the explanatory variables is

(15) $$-\frac{n_{p}}{n_{c}-n_{p}}\frac{\gamma }{\lambda_{*}}.$$

where *γ* is the effect of *x*_*i*_ on *y*_*i*_, and λ_*_ is the coefficient on ${\overset{¯}{x}}_{-i}^{p}$ from an OLS regression of ${\overset{¯}{y}}_{-i}^{p}$ on ${\overset{¯}{x}}_{-i}^{p}$ and the dummy variables for each of the clusters **d**_*i*_; i.e. the first stage in a two‐stage least squares procedure.

The proof is given in Appendix B. The above proposition shows that the asymptotic bias is inversely related to the effect of the instrument on the peers’ average outcome, λ_*_, and converges to zero if $n_{c}$ tends to infinite as long as $n_{p}$ remains bounded.^11^ Similarly, the larger the peer group, $n_{p}$, the larger the bias. Notice that Assumption A2 implies that the size of peer groups is smaller than the size of the clusters and this ensures that the bias does not explode. In the case where there is just one cluster i.e. $n_{c}=N$, we have random allocation of peers across individuals and the asymptotic bias goes to zero for *N* which tends to ∞.

Note that *IV approach 0* and *1* can easily be adjusted to account for additional explanatory variables, by extending model (12) to include covariates, say, $Q_{*}$. The asymptotic results can be extended to this case by applying the Frisch–Waugh–Lovell theorem which implies replacing y_*_ with the residual of the regression of y_*_ on $Q_{*}$, i.e. $M_{Q_{*}}y=\left[I-Q_{*}{\left(Q_{*}^{\prime }Q_{*}\right)}^{-1}Q_{*}^{\prime }\right]y_{*}$ and similarly replacing (**Wy**)_*_ with $\left(M_{Q_{*}}{\mathrm{Wy}}_{*}\right)$ and $X_{*}$ with $\left(M_{Q_{*}}X_{*}\right)$. The conclusions remain unchanged, i.e. *IV approach 1* provides a consistent estimation for the peer effect *ρ*, while *IV approach 0* is inconsistent.

## 3 A brief discussion of the literature

Although we recognize that most empirical peer effects estimations include the instrument at the individual level, some papers have not. For example, Kang (2007) examines peer effects in students’ maths attainment, estimating a school fixed effects model that uses peers’ average science scores to instrument for peers’ average maths scores, but excludes the individual's science score from the structural equation. Hence, despite students being quasi‐randomly allocated from elementary to middle schools, not including the instrument at the individual level, combined with the inclusion of school fixed effects, leads to biased peer effects estimates. Similarly, Figlio (2007) investigates peer effects in students’ disruptive behaviour, using the proportion of classroom boys with girls’ names to instrument for peers’ average behaviour, while adjusting for individual and grade fixed effects, but not including an indicator whether the individuals themselves have a girls’ name. Lundborg (2006) investigates peer effects in adolescent substance use, estimating school‐grade fixed effects models that use various peer‐level instruments, several of which are excluded at the individual‐level from the structural equation. For example, one of the instruments for peer average illicit drug use is the proportion of peers who indicate they know someone who could give or sell them drugs; and one of the instruments for peer average binge drinking is the proportion of peers who indicate their parents would provide beer if asked. These variables, however, are not included at the individual level.

As we discuss above, it is difficult to predict the sign and magnitude of the asymptotic bias as it depends on different factors. Nevertheless, we can comment on this to an extent. Equation (15) shows that the asymptotic bias has the same sign as $-\frac{\gamma }{\lambda_{*}}$. Because it is generally true that the relationship between *x* and *y* at the peer group level also holds at the individual level, *γ* and λ_*_ are of the same sign, implying the bias is negative. Furthermore, the magnitude of the asymptotic bias depends on the ratio $\frac{n_{p}}{n_{c}-n_{p}}$. This suggests that in primary school settings, which tend to be smaller than secondary schools but with similar class sizes, one would expect to see larger biases if classes are defined as the peer group, all else equal.

As an example, consider the study by Kang (2007). Their data include 4,813 students in 248 classes and 124 schools, suggesting that the average peer group (i.e. class) and school include 19 and 39 pupils respectively. The estimated λ_*_ (i.e. the effect of the instrument in the first stage) is 0.64. If we assume that λ_*_≈*γ* (i.e. the effect of the instrument at the individual level on the individual outcome is similar to the first stage), the asymptotic bias approximates $-\frac{n_{p}}{n_{c}-n_{p}}\frac{\gamma }{\lambda_{*}}=-0.95\times 1=-0.95$.^12^ This suggests that the bias may be relatively large, indicating that it does matter whether the instrument at the individual level is included as a covariate or not. Their peer effect is estimated to be around 0.3. Our back‐of‐the envelope calculations suggest that this is an underestimate, with our estimate closer to 1.25. Although this is a large difference, we cannot comment on its significance. Furthermore, we note that the bias also depends on the extent to which our assumptions, listed in the proposition above, hold. Indeed, it relies on the true model being defined by equation (12), in the sense that *x*_*i*_ enters the equation in an additively separable way, which may not be the case. Similarly, we assume that each individual has the same number of peers and the same number of cluster members, which is unlikely to be the case. The true data structure will therefore also impact on the estimate of the bias.

## 4 Conclusion

A popular approach to estimating peer effects in the economics literature is to fit linear in mean regressions of individuals’ outcomes on the corresponding average outcomes of their peers. A common approach to deal with the simultaneity of the peer effect is to use IV, instrumenting the average outcome of peers with the peer average of certain characteristics. We show that the validity of the relevance assumption in this setting has a subtle, but important implication: the instrument at the individual level must be included as an additional explanatory variable. We show that failing to do so leads to biased and inconsistent peer effect estimates. We demonstrate that the only case when consistency holds, is if peers are randomly allocated across individuals. However, even then, the IV estimation remains inconsistent if the model includes cluster fixed effects in addition to the peer effect. Examples are those where randomization takes place within, but not across, schools or neighbourhoods, where the inclusion of school or neighbourhood fixed effects (a necessity as randomization takes place within these clusters) renders the estimates inconsistent. In that case, the bias is induced by the inclusion of cluster fixed effects and its within‐cluster transformation; something that has hitherto not been discussed in this literature. We present a simple solution: the instrument at the individual level must be included in the peer effects model. This leads to consistent peer effect parameter estimates under the assumptions required for IV.

## Notes

1 Different types of instruments have been used, including, (i) the average price of peers’ decisions which is exogenously shifted by the introduction of policy or programme affecting only some of the people (see the ‘partial‐population’ identification approach defined by Moffitt (2001), and the application in Dahl, Loken and Mogstad (2014)); (ii) peer averages of predetermined variables that affect peers but only influence the individual outcome through the peers’ outcome (e.g. O’Malley *et al*., 2014), (iii) average characteristics of peers, who are not direct peers (see Bramoullé, Djebbari and Fortin, 2009; De Giorgi, Pelizzari and Redaelli, 2010; Nicoletti and Rabe, 2016; Nicoletti, Salvanes and Tominey, 2016). Other approaches to identify peer effects include (natural) experiments (e.g. Hoxby, 2000; Duflo and Saez, 2003; Gould and Winter, 2009), random allocation of peers (e.g. Sacerdote, 2001; Kremer and Levy, 2008), and fixed effects, value‐added approaches (e.g. Neidell and Waldfogel, 2010).

2 Boozer and Cacciola (2001) and Angrist (2014) additionally show that the individual variable, say *x*, whose peer average, $\overset{¯}{x}$, is used to instrument the peer average outcome must have some variation *within* as well as *between* peer groups.

3 To avoid confusion with ‘peer groups’, we refer to these (often larger) groupings such as schools or neighbourhoods as ‘clusters’.

4
**W** is generally constructed to have zero elements on the leading diagonal, ensuring that **Wy** excludes the individuals themselves. We also assume that the peer relationships be symmetric, so that **W** is symmetric.

5 Appendix A shows this is true under plausible assumptions.

6 Here, we follow the existing literature that almost exclusively considers specifications in which all covariates enter additively and linearly (including the literature that *does* account for the instrument at the individual level; section 3 discusses some of the relevant literature). We use this specification when deriving the asymptotic bias below. However, we note that these derivations do not generalize to situations where the true model includes some other function of the instrument at the individual level (e.g. **X** ^**2**^ or **ln(X)**). Hence, in such cases, the asymptotic bias is also likely to be different. Nevertheless, because the majority of studies specify the model as in equation (3), we derive the bias for this specification.

7 Note the difference with ${\hat{\rho }}_{IV0}$ in equation (2), i.e. the cluster–mean deviation, indicated by *.

8 The idea is similar to the ‘Nickell bias’ (Nickell, 1981) in dynamic models that include individual fixed effects, leading to a correlation between the lagged‐dependent variable and the mean deviation of the error term. However, the Nickell bias reduces as the number of time periods increases, the bias of ${\hat{\rho }}_{*IV0}$ reduces as the cluster size increases relative to the peer group, since the contribution of each peer to the cluster means becomes negligible.

9 Although the instrument at the individual level has to be included as an additional explanatory variable, the form in which it enters matters for the bias. As the existing literature mainly considers additively separable specifications, we characterize the bias for this case only in section ‘Asymptotic bias’.

10 In addition to avoiding the bias discussed here, it also corrects for the ‘exclusion bias’ defined by Caeyers and Fafchamps (2016).

11 The latter also holds for the ‘exclusion bias’, which Caeyers and Fafchamps (2016) show converges to zero when $n_{c}$ tends to infinite while $n_{p}$ remains bounded.

12 We do not know the true value of *γ*, as this is precisely the parameter that is not estimated. In our illustrative application, presented in the Web Appendix, the ratio $\frac{\gamma }{\lambda_{*}}=\frac{0.332}{0.290}=1.145$. Hence, although this is tentative as this estimate is obtained from a different data set, it suggests that assuming *γ*=*λ*_*_ is a reasonable approximation. It is difficult to characterize the likely bias in Figlio (2007) and Lundborg (2006); their data contain approximately 76,000 and 3,000 students respectively, but they do not mention how many schools and classrooms they observe, and Lundborg (2006) does not report the first stage estimates.

## Appendix A Proof by contradiction

In the following, we prove that, if the instrumental variables **WX** satisfy the relevance and exclusion conditions for the estimation of the peer effect in model (1), then **X** directly affects **y**, and hence model (1) is misspecified because it omits **X** from the explanatory variables. The proof does not rely on any specific type of peer assignment.

As used in the spatial statistics and econometrics literature on peer effect (see e.g. Lee, 2007; Bramoullé *et al*., 2009), we can derive the reduced form of model (1),

(A1) $$y={\left(I-W\rho \right)}^{-1}u,$$

where **I** is the identity matrix of size *N* and we assume that |*ρ*|<1 and *ρ*>0 so that the matrix (**I**−**W** *ρ*) is invertible and the peer effect is positive. By using the series expansion ${\left(I-W\rho \right)}^{-1}=\sum_{s=1}^{\infty }\rho^{s}W^{s}$ we can then rewrite the reduced form model as

(A2) $$y=\sum_{s=1}^{\infty }\rho^{s}W^{s}u.$$

Given equation (A2), the symmetry of the matrix **W** (because of the symmetry of peer relationships), and the fact that all variables are demeaned, we can prove that the covariance between **Wy** and **WX** is

(A3) $$Cov\left({\mathrm{Wy}}^{\prime },\mathrm{WX}\right)=E\left(\sum_{s=1}^{\infty }\rho^{s}u^{\prime }W^{s+2}X\right).$$

This implies that **WX** are relevant instruments for **Wy** only if the right‐hand side of the above equation is different from zero. We can rewrite this as a sum of expectations, with weights given by *ρ* ^*s*^:

(A4) $$\sum_{s=1}^{\infty }\rho^{s}E\left(u^{\prime }W^{s+2}X\right).$$

Because *ρ* ^*s*^>0, the above expression is different from zero if at least one of the following conditions hold: (i) **u** depends linearly on **WX**; (ii) **u** depends linearly on **W** ^*h*^ **X** for some *h*>1 but does not depend linearly on **WX**; (iii) **u** depends linearly on **X**. Condition (i) would invalidate the instrumental variable because the exclusion restriction would not be satisfied. Condition (ii) would imply that the outcome **y** depends on the average of **X** for peers separated by *h* interactions^13^ but not on the average of **X** for direct peers (i.e. peers separated by 1 interaction). This is unlikely, as it is implausible that peers separated by more than one interaction have a larger influence on the outcome **y** than direct peers. This implies that condition (iii) must hold to guarantee that the right‐hand side of equation (A3) be non‐zero. In other words, **X** and **u** are correlated, implying that **X** are omitted variables. The only situation when omitting **X** would not bias the estimation of the peer effect is when there is no correlation between the instruments **WX** and **X**.

## Appendix B Proof of Proposition 1

While the true model is given by model (14) (see Assumption A1), the estimation model omits the explanatory variable *x*_*i*_ and is given by

(B1) $$y_{i}={\overset{¯}{y}}_{-i}^{p}\rho +d_{i}\delta +\nu_{i},$$

where *i*=1,…,*N* and the error term *ν*_*i*_=*x*_*i*_ *γ*+*e*_*i*_. Notice that model (B1) is identical to model (8), but it is expressed as a set of *N* individual equations rather than in matrix notation. To control for the cluster effect, we can transform all variables in model (B1) using within‐cluster deviations:

(B2) $$y_{i}-{\overset{¯}{y}}_{i}^{c}=\left({\overset{¯}{y}}_{-i}^{p}-{\overset{¯}{\overset{¯}{y}}}_{i}^{\mathrm{pc}}\right)\rho +\nu_{i}-{\overset{¯}{\nu }}_{i}^{c},$$

where ${\overset{¯}{y}}_{i}^{c}$ and ${\overset{¯}{\nu }}_{i}^{c}$ are the averages of *y*_*i*_ and *ν*_*i*_ across all members belonging to the same cluster as individual *i* and, similarly, ${\overset{¯}{\overset{¯}{y}}}_{i}^{\mathrm{pc}}=\sum_{j=1}^{n_{c}}{\overset{¯}{y}}_{-j}^{p}/n_{c}$ is the cluster average of the peer average of all members belonging to the same cluster as individual *i*. Note that (**Wy**)_*_, **y**_*_ and (**WX**)_*_ defined in section ‘The case with cluster fixed effects’ are equivalent to the vectors of the individual within‐cluster deviations $\left({\overset{¯}{y}}_{-i}^{p}-{\overset{¯}{\overset{¯}{y}}}_{i}^{\mathrm{pc}}\right)$, $\left(y_{i}-{\overset{¯}{y}}_{i}^{c}\right)$ and $\left({\overset{¯}{x}}_{-i}^{p}-{\overset{¯}{\overset{¯}{x}}}_{i}^{\mathrm{pc}}\right)$ respectively. Note also that the IV estimator of the peer effect *ρ* based on the misspecified model (B2), which instruments $\left({\overset{¯}{y}}_{-i}^{p}-{\overset{¯}{\overset{¯}{y}}}_{i}^{\mathrm{pc}}\right)$ with $\left({\overset{¯}{x}}_{-i}^{p}-{\overset{¯}{\overset{¯}{x}}}_{i}^{\mathrm{pc}}\right)$, is equivalent under Assumption A3/A4 to that defined in (B1):

(B3) $$p-\text{lim}\;{\hat{\rho }}_{*IV0}=\rho +{\left[\lambda_{*}^{\prime }E\left({\left(\mathrm{WX}\right)}_{*}^{\prime }{\left(\mathrm{WX}\right)}_{*}\right)\lambda_{*}\right]}^{-1}\lambda_{*}^{\prime }E\left({\left(\mathrm{WX}\right)}_{*}^{\prime }\left(X_{*}\gamma +e_{*}\right)\right),$$

where $\lambda_{*}=p-\text{lim}{\left({\left(\mathrm{WX}\right)}_{*}^{\prime }{\left(\mathrm{WX}\right)}_{*}\right)}^{-1}{\left(\mathrm{WX}\right)}_{*}^{\prime }{\left(\mathrm{Wy}\right)}_{*}$ is the coefficient on ${\overset{¯}{x}}_{-i}^{p}$ in the first stage regression of ${\overset{¯}{y}}_{-i}^{p}$ on ${\overset{¯}{x}}_{-i}^{p}$ and the cluster dummy variables, **d**_*i*_, and ***γ*** is the effect of *x*_*i*_ in the true model [Link]. Notice that because the explanatory variable *x*_*i*_ and the instrument ${\overset{¯}{x}}_{-i}^{p}$ are univariate variables, the coefficients ***λ***_*_ and ***γ*** are actually scalars, which we denote as *λ*_*_ and *γ*. Under the assumption of exogeneity of the instrument (Assumption A4), $E({\left(\mathrm{WX}\right)}_{*}^{\prime }e_{*})=0$ so that the asymptotic bias becomes:

(B4) $$p-\text{lim}\;{\hat{\rho }}_{*IV0}-\rho ={\left[\lambda_{*}^{\prime }E\left({\left(\mathrm{WX}\right)}_{*}^{\prime }{\left(\mathrm{WX}\right)}_{*}\right)\lambda_{*}\right]}^{-1}\lambda_{*}^{\prime }E\left({\left(\mathrm{WX}\right)}_{*}^{\prime }\left(X_{*}\gamma \right)\right).$$

Because we assume that each individual has the same number of peers $n_{p}$ and all his/her peers belong to the same cluster (see Assumption A2), ${\overset{¯}{\overset{¯}{x}}}_{i}^{\mathrm{pc}}=\left(\sum_{j=1}^{n_{c}}\sum_{s=1,\;s\neq j}^{n_{p}}x_{s}\right)/\left(n_{c}n_{p}\right)={\overset{¯}{x}}_{i}^{c}$. The intuition here is that the characteristic *x*_*k*_ of individual *k* belonging to the same cluster as individual *i* appears $n_{p}$ times in the sum of the numerator of $\left[\left(\sum_{j=1}^{n_{c}}\sum_{s=1,\;s\neq j}^{n_{p}}x_{s}\right)/\left(n_{c}n_{p}\right)\right]$ as a peer of her $n_{p}$ peers. This implies that

$$\left(\sum_{j=1}^{n_{c}}\sum_{s=1,\;s\neq j}^{n_{p}}x_{s}\right)/\left(n_{c}n_{p}\right)=\left(\sum_{j=1}^{n_{c}}x_{j}n_{p}\right)/\left(n_{c}n_{p}\right)=\sum_{j=1}^{n_{c}}{\overset{¯}{x}}_{j}/n_{c}={\overset{¯}{x}}_{i}^{c}.$$

Because all variables are demeaned, *x*_*i*_ is i.i.d. across individuals (see Assumption A1) and peers are randomly allocated across individuals within clusters (Assumption A3), $E({\left(\mathrm{WX}\right)}_{*}^{\prime }X_{*})$ is the covariance between $\left({\overset{¯}{x}}_{-i}^{p}-{\overset{¯}{x}}_{i}^{c}\right)$, and $\left(x_{i}-{\overset{¯}{x}}_{i}^{c}\right)$ and $E({\left(\mathrm{WX}\right)}_{*}^{\prime }{\left(\mathrm{WX}\right)}_{*})$ is the variance of $\left({\overset{¯}{x}}_{-i}^{p}-{\overset{¯}{x}}_{i}^{c}\right)$. Hence, equation (B3) can be rewritten as

(B5) $$p-\text{lim}\;{\hat{\rho }}_{*IV0}-\rho =Cov\left({\overset{¯}{x}}_{-i}^{p}-{\overset{¯}{x}}_{i}^{c},x_{i}-{\overset{¯}{x}}_{i}^{c}\right)Var{\left({\overset{¯}{x}}_{-i}^{p}-{\overset{¯}{x}}_{i}^{c}\right)}^{-1}\frac{\gamma }{\lambda_{*}}.$$

We can prove that

(B6) $$\begin{array}{cc} Cov(\left({\overset{¯}{x}}_{-i}^{p}-{\overset{¯}{x}}_{i}^{c}\right),\left(x_{i}-{\overset{¯}{x}}_{i}^{c}\right)) & =Cov\left({\overset{¯}{x}}_{-i}^{p},x_{i}\right)-Cov\left({\overset{¯}{x}}_{-i}^{p},{\overset{¯}{x}}_{i}^{c}\right)-Cov\left({\overset{¯}{x}}_{i}^{c},x_{i}\right)+Var\left({\overset{¯}{x}}_{i}^{c}\right)\\ & =0-\frac{\sigma_{x}^{2}}{n_{c}}-\frac{\sigma_{x}^{2}}{n_{c}}+\frac{\sigma_{x}^{2}}{n_{c}}=-\frac{\sigma_{x}^{2}}{n_{c}}. \end{array}$$

by using the following conditions

- *x*_*i*_ is i.i.d. across individuals with mean zero and variance $\sigma_{x}^{2}$ (see Assumption A1);
- peers are randomly allocated across individuals within clusters (see Assumption A3);
- all peers of members of a cluster belong to the same cluster (see Assumption A2).
- - Conditions (i) and (ii) implies that *x*_*i*_ is uncorrelated with ${\overset{¯}{x}}_{-i}^{p}$ so that $Cov({\overset{¯}{x}}_{-i}^{p},x_{i})=0$.
  - Using assumptions (i) and (iii),
    $$Cov\left({\overset{¯}{x}}_{-i}^{p},{\overset{¯}{x}}_{i}^{c}\right)=Cov\left(\sum_{j=1,\;j\neq i}^{n_{p}}x_{j},\sum_{s=1}^{n_{c}}x_{s}\right)/\left(n_{c}n_{p}\right)=E\left(\sum_{j=1,\;j\neq i}^{n_{p}}x_{j}^{2}\right)/\left(n_{c}n_{p}\right)=\sigma_{x}^{2}/n_{c}$$
  - Because *x*_*i*_ is included in the cluster average, ${\overset{¯}{x}}_{i}^{c}$,
    $$Cov\left({\overset{¯}{x}}_{i}^{c},x_{i}\right)=Cov\left(\sum_{s=1}^{n_{c}}x_{s},x_{i}\right)/n_{c}=\sigma_{x}^{2}/n_{c}.$$
  - Finally, using condition (i), $Var\left({\overset{¯}{x}}_{i}^{c}\right)=\frac{\sigma_{x}^{2}}{n_{c}}$.

Using the same reasoning, we can show that

(B7) $$Var\left({\overset{¯}{x}}_{-i}^{p}-{\overset{¯}{x}}_{i}^{c}\right)=Var\left({\overset{¯}{x}}_{-i}^{p}\right)+Var\left({\overset{¯}{x}}_{i}^{c}\right)-2Cov\left({\overset{¯}{x}}_{-i}^{p},{\overset{¯}{x}}_{i}^{c}\right)=\frac{\sigma_{x}^{2}}{n_{p}}+\frac{\sigma_{x}^{2}}{n_{c}}-2\frac{\sigma_{x}^{2}}{n_{c}}=\sigma_{x}^{2}\frac{n_{c}-n_{p}}{n_{c}n_{p}}.$$

Replacing $Cov(\left({\overset{¯}{x}}_{-i}^{p}-{\overset{¯}{x}}_{i}^{c}\right),\left(x_{i}-{\overset{¯}{x}}_{i}^{c}\right))$ and $Var({\overset{¯}{x}}_{-i}^{p}-{\overset{¯}{x}}_{i}^{c})$ in equation (B5) with the last right hand side terms in equations (B6) and (B7), we get

(B8) $$p-\text{lim}\;{\hat{\rho }}_{*IV0}-\rho =-\frac{n_{p}}{n_{c}-n_{p}}\frac{\gamma }{\lambda_{*}}.$$
